# Why does pulsed laser-deposited amorphous NiO_*x*_ serve as an excellent electrode material for revolutionizing glucose detection?

**DOI:** 10.1039/d5ra09463a

**Published:** 2026-02-26

**Authors:** Akshay Parab, Suhas M. Jejurikar, Jash Salunke, Dattatray Late

**Affiliations:** a National Centre for Nanosciences and Nanotechnology, University of Mumbai, Kalina Campus Santacruz (E) Mumbai-400098 India jejusuhas@gmail.com; b University of Technology of Troyes (UTT) 12 Rue Marie Curie, 42060 CS, Cedex 10004 Troyes France; c Department of Physics, Federal University of Lavras, Campus Universitário PO Box 3037 Lavras MG Brazil; d Universal AI University Kushivili PO Karjat (Near Mumbai) Maharashtra 410201 India

## Abstract

Amorphous materials offer distinct properties, such as specific electronic states, a multitude of surface dangling bonds, unsaturated coordination, and enhanced charge transfer, that may improve their electrochemical performance compared with their nano and bulk counterparts. Given their intriguing characteristics, herein, we report a revolutionary electrochemical response of amorphous NiO_*x*_ to glucose, which is confirmed through cyclic voltammetry measurements in an alkaline medium. To investigate this, amorphous NiO_*x*_ was grown on a commercially available screen-printed electrode using pulsed laser deposition. The amorphous nature of NiO_*x*_ was confirmed using transmission electron microscopy, while nickel with multi valances was confirmed using an X-ray photoelectron microscopy. As the glucose concentration varied in the alkaline medium, a negligible change in current values is observed, whereas a systematic change is observed in oxidation potential *E*_p_ in the CV plot. The diffusion coefficient estimated by fitting the experimental data to the Randles–Sevcik expression is observed to be one order higher than that of the reported NiO_*x*_ electrodes. The negligible change in current values and the high diffusion coefficient observed herein indicate a charge transfer process that might occur on the electrode surface. However, changes observed in the oxidation potential indicate a surface concentration of active species/phases (*viz.* α-Ni(OH)_2_, β-Ni(OH)_2_, β-NiOOH and γ-NiOOH) that drives the electrochemical reactions responsible for glucose oxidation. Various mechanisms proposed herein based on the theoretical models suggest that the surface modifications of the electrode material are a first-order process that gradually evolve due to the formation of multiple phases, *i.e.*, the formation of various nickel hydroxide species.

## Abstract 2

Amorphous materials offer distinct properties *viz.* specific electronic states, multitude of surface dangling bonds, unsaturated coordination, enhanced charge transfer that may improve electrochemical performance compared to their other counterparts.

## Graphical abstract

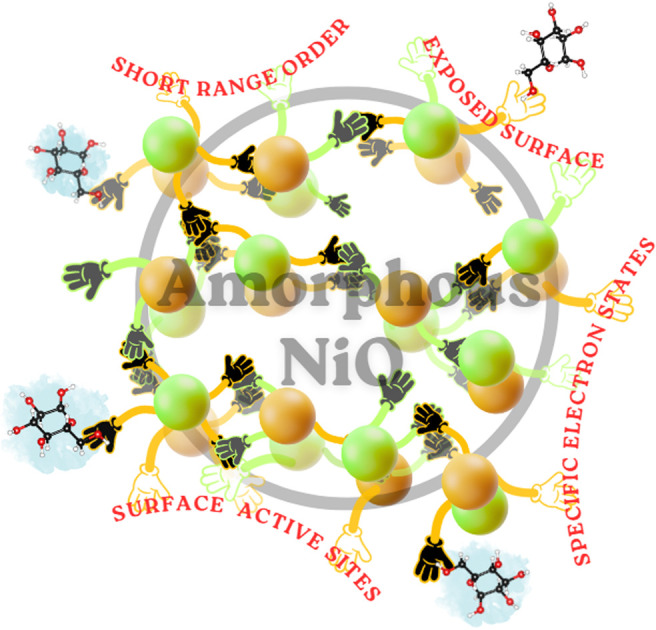

## Introduction

**Fig. 1:**
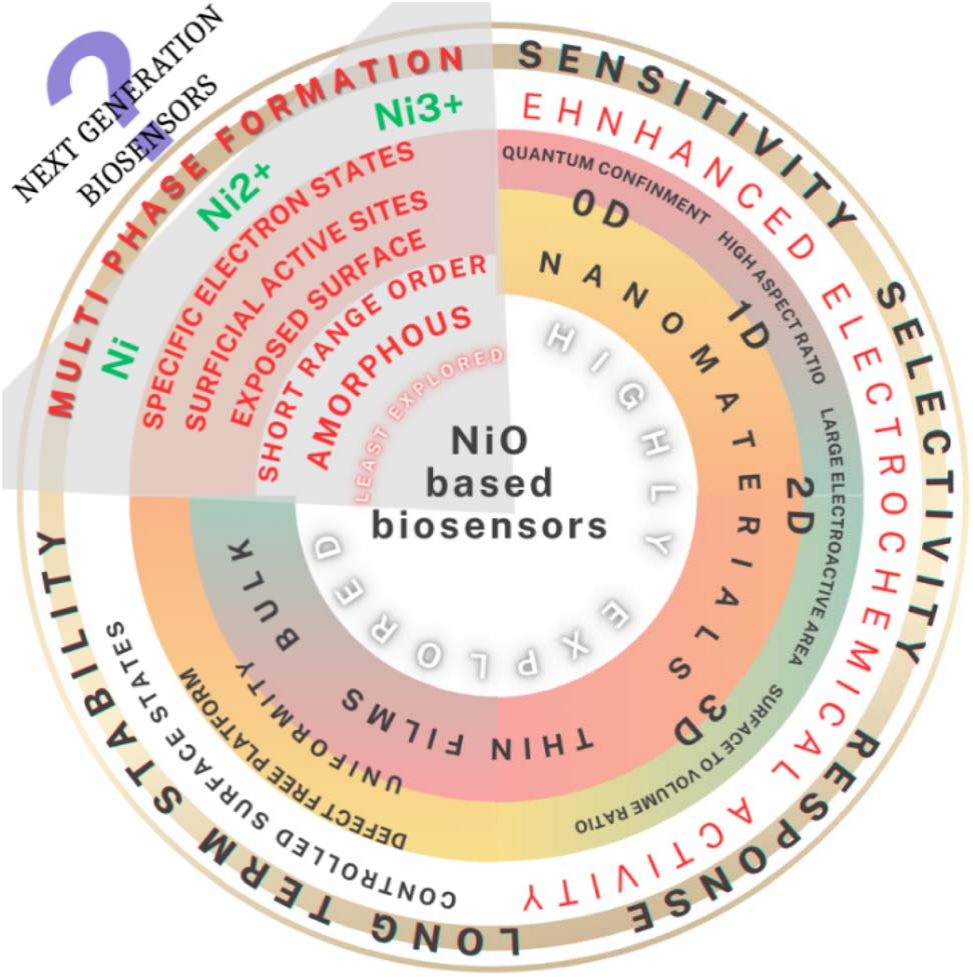
General relationships between NiO in its various forms, *i.e.* bulk, nanostructures and amorphous, and their properties.

Diabetes, a chronic disease that affects millions of people worldwide, has been declared a “silent epidemic”.^1^ The disease caused by a metabolic disorder is examined by monitoring the glucose level (for adults without diabetes, it is typically 72–99 mg dL^−1^, while post-meal levels may vary from 80 to 130 mg dL^−1^) present in human body fluids to determine appropriate treatments.^2^ Therefore it is a necessity for improving the available infrastructure to detect glucose levels in body fluids in a reliable manner, easy to operate and, more importantly, affordable.^3^ Thus, this requirement has revolutionized the shift from conventional detection techniques used to monitor blood glucose to the self-monitoring of blood glucose using handy, accurate, easy-to-operate and affordable glucometers (point-of-care devices).^4,5^ Most glucometers require specific test strips with enzymatic and non-enzymatic platforms. Enzyme-based test strips, which are expensive and have a shorter with shelf-life, use enzymes as active materials to generate responses upon the detection of a specific biomolecule.^3^ Alternatively, non-enzymatic test strips have been developed as alternatives to enzyme-based platforms by leveraging the electron transfer properties of various metals and/or metal oxides. Metals such as copper (Cu), nickel (Ni), platinum (Pt), magnesium (Mg), and gold (Au)^2,3^ and metal oxides such as CuO, CoO, MnO_2_, ZnO, and NiO^6^ are used as catalysts for glucose oxidation, mostly converting it into gluconolactone or gluconic acid.^7^ To target glucose diffused in human body fluids, including urine, tears, blood, and saliva, it is important to improve the sensitivity and selectivity of these platforms.^8^ To enhance electrochemical activities, researchers have used various forms of these materials, including thin films^9^ and nanostructures ranging from zero- to three-dimensions,^10^ which possess a high surface-to-volume ratio, good conductivities, shock-bearing abilities, color tenability and effective electron transfer.^7,9,11,12^ Among such materials, previous research on NiO has demonstrated its excellent catalytic, magnetic, electrochromic, low toxicity, biocompatibility, and optical and electrochemical properties.^10,13,14^ Further, the isoelectric point of NiO (10.8), which is higher than that of other metal oxides, serves as an excellent matrix for improved immobilization of biomolecules.^15^ In addition, its excellent chemical stability and considerable higher electro-catalytic and electron transfer properties have been reported to enhance the intensity of electrochemical signals suitable for the detection of analytes at very low concentrations.^10,13,16–18^ NiO possesses multi valence states (Ni^2+^ and Ni^3+^ due to the complex electron configuration, allowing it to take different oxidation states), where the dominant presence of the valence state, *i.e.*, Ni^2+^, is responsible for facilitating effective electron transfer, which is essential for the oxidation of glucose molecules.^12,19^ This makes it fascinating to synthesize NiO with mixed valence states. This fact thus motivates the growth of NiO, which has multi valances. Today, the strategic phase engineering of nanomaterials has enabled researchers to tune a variety of their nanocrystalline structures and properties.^20^ This has resulted in the broadening of their applications in a variety of fields. However, the amorphous counterparts of these materials, which remain the least studied have recently received attention.^14,21^ The general relationships between NiO in its various forms, *i.e.* bulk, nanostructures and amorphous, and their properties are illustrated in Fig. 1. Amorphous NiO possesses unique structural features, such as disordered atomic structure, unsaturated coordination, surface dangling bond, and specific electron state. The amorphous form thus produces more active sites, can easily recombine with other surrounding units *via* super-strong interfacial interactions and can optimize the reaction pathways to enhance the intrinsic activity, which is essential for sensing biomolecules *via* electrochemistry.

In this article, we report the synthesis of amorphous NiO_*x*_ on a commercially available screen-printed electrode using pulsed laser deposition (PLD). This study has two clear aims. The first is to confirm the presence of a multi valence nature associated with amorphous NiO_*x*_ grown on screen-printed paper; electrode characterization techniques, namely transmission electron microscopy and X-ray photoelectron spectroscopy, were used. The second is to address the electrochemical process responsible for the oxidation of the glucose molecule due to amorphous NiO_*x*_ deposited on the electrode, for which a cyclic voltammogram was recorded against the glucose molecule in an alkaline medium at a scan rate. Here, we consider various mechanisms proposed by researchers to confirm the electron transfer that takes place between multi valances of Ni and is responsible for oxidizing a glucose molecule.

## Experimental

Screen-printed electrodes (Make: Zensor, working area 3 mm) with carbon as the counter electrode as well as the working electrode and Ag as the reference electrode were used for the proposed study. The working area of the electrode was coated with amorphous NiO_*x*_ using pulsed laser deposition. The KrF excimer laser (*λ* = 248 nm, energy density at target surface = 2 J cm^−2^, pulse repetition rate = 5 Hz, and duration = 5 min) was used as an ablation source, and an NiO target (target prepared by solid-state route at 1200 °C using high-purity powder procured from Sigma-Aldrich-99.99%, product code: 203882) was employed. To ensure uniform coating of NiO_*x*_ only on the working area of the electrode, the masked electrode was fixed on a substrate holder and placed inside a deposition chamber at a distance of 4.5 cm parallel to the target surface. The chamber was then evacuated to a base pressure of ∼10^−6^ torr using a turbo molecular pump backed up by a scroll pump. To ensure the presence of multi-valance Ni in NiO_*x*_, depositions were carried out in the presence of argon gas (purity 99.999%), maintaining the chamber pressure at 1 × 10^−4^ torr. To confirm the morphological and structural properties of the NiO_*x*_ layer deposited herein, a transmission electron microscope (TEM, Make: FEI, Model: Technai, Accelerating Voltage: 300 keV) was extensively used. To prepare the sample for TEM, a NiO_*x*_ layer was deposited on a glass substrate under similar growth conditions. The deposits on the glass substrate were then scratched out using a Teflon spatula and loaded onto the carbon-coated TEM grid. The chemical composition of the film was studied using X-ray photoelectron spectroscopy (XPS), for which an X-ray photoelectron spectrometer (Make: SPECS Surface Nano Analysis GmbH, Germany) had Al K_α_ (1486.6 eV) as a source and instrument calibrated with an Au 4f_7/2_ line positioned at 83.8 eV was used. Before recording any scan, the C 1s line positioned at 284.86 eV was recorded to confirm the charging effect, if any. The spectra were analysed by deconvoluting high resolution scans using XPS peak 4.1 with Shirley type baseline and a mixed Gaussian–Lorentzian line shape profile. A cyclic voltammogram (CV) was recorded to study the electrochemical response of these electrodes using a potentiostat (Make: CHI600E workstation) in alkaline medium (0.1 M NaOH). To confirm the sensitivity of the electrodes against glucose molecules and their reproducibility, sets of such electrodes were prepared and tested by recording CV measurements optimized at a scan rate of 20 mV s^−1^. For this, a stock solution (100 mM) of glucose was prepared in 100 ml of DI. The glucose concentration in the alkaline medium, namely 5–500 µm, was prepared using a stock solution. After each measurement, the electrodes were thoroughly washed in DI water, and freshly prepared electrolytes were used before proceeding to the next measurement.

## Results and discussion

**Fig. 2:**
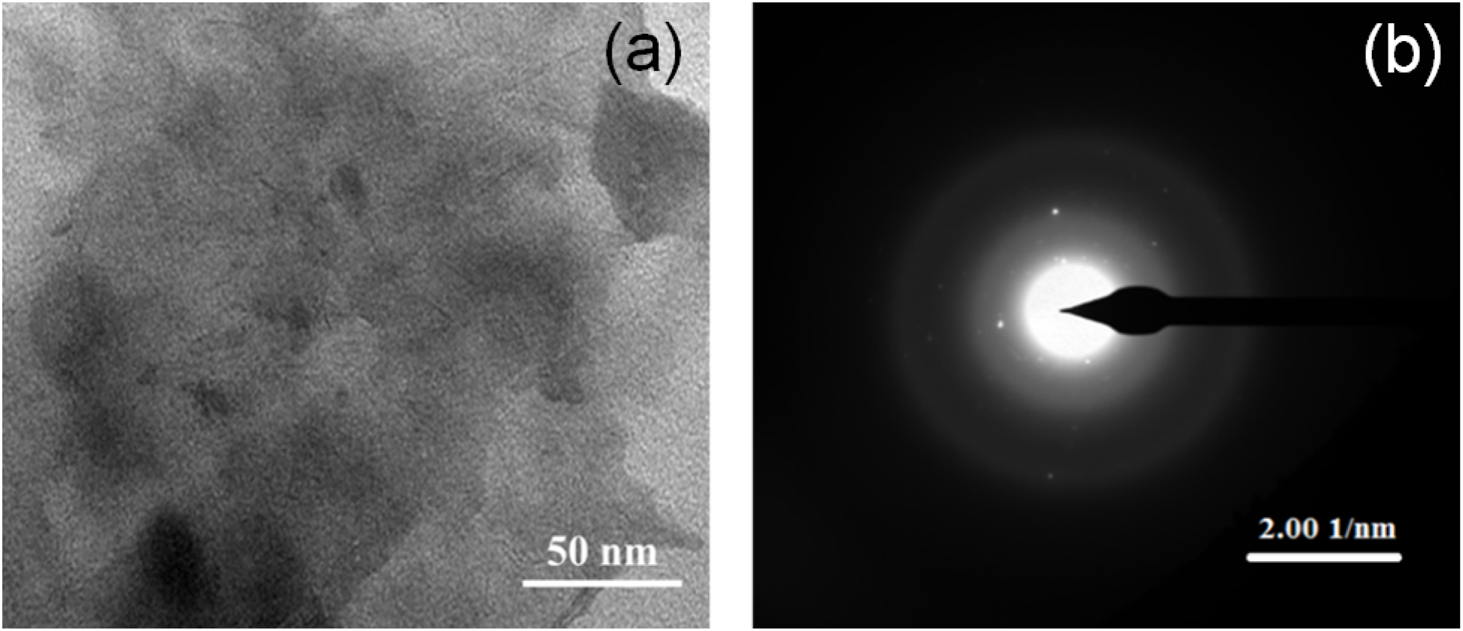
(a) TEM image showing the morphology of NiO_*x*_ and (b) SAED pattern revealing structural characteristics of NiO_*x*_.

Fig. 2 presents the morphological and structural investigations performed on the NiO_*x*_ specimen using TEM in multiple places. Fig. 2a shows one of the TEM images recorded. The absence of any kind of crystalline form or any kind of atomic arrangement in the image clearly indicates the amorphous nature of the specimen.

To ensure the same, structural investigations were also performed on the specimen by recording selected area electron diffraction (SAED) patterns at multiple places on the TEM grid. Fig. 2b shows one of the SAED patterns recorded. The diffused ring patterns observed herein support the claim made about the amorphous nature of deposits. The elemental composition of the deposits was confirmed by recording the XPS spectra. Prior to the XPS measurements, NiO_*x*_ was deposited on the screen-printed electrode under the same deposition conditions.

**Fig. 3:**
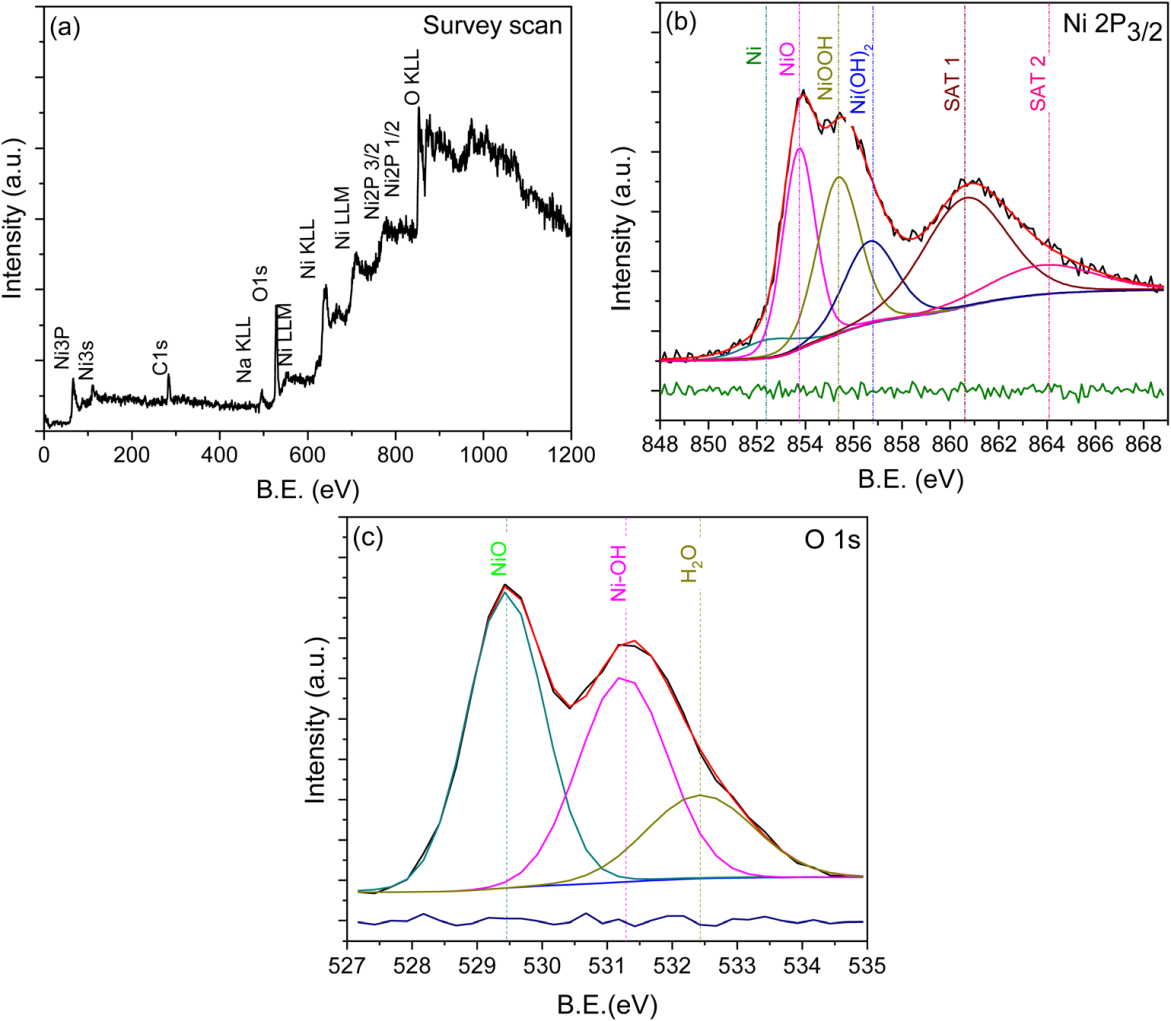
Elemental analysis of the NiO_*x*_ sample using XPS: (a) survey scan and high-resolution elemental scans of (b) Ni 2p_3/2_ and (c) O 1s.

Fig. 3a presents the XPS survey scan recorded on the specimen showing the presence of only elements, namely Ni, O and C, confirming the absence of any other impurities in the specimen. To confirm the multiple charge states of the observed elements, high-resolution XPS spectra were also recorded. Accordingly, Fig. 3b presents the high resolution XPS spectrum of Ni 2p, which shows a region, namely Ni 2p_3/2_ (848–868 eV), due to spin orbit coupling. Each spectrum is deconvoluted into multiple peaks due to its asymmetric nature. The presence of metallic Ni in the specimen is realized from the deconvoluted peaks that are positioned at 852.5 eV. Other deconvoluted peaks positioned between 854 and 857 eV in Fig. 3b can be attributed to the multiple valances/charge states of Ni, corresponding to the formation of NiO, NiOOH and Ni(OH)_2_.^14,21^ However, the rest of the peaks observed at higher binding energies in both spectra are confirmed to be satellite peaks.^22,23^ Similarly, the O 1s spectrum is deconvoluted into multiple peaks due to its asymmetric nature. The peak positioned at ∼529.5 eV can be assigned to the lattice Ni bound with oxygen, confirming the octahedral bonding of NiO,^24^ while the remaining peaks positioned at 531.25 and 532.50 eV can be attributed to the Ni bound with OH and water molecules, respectively.^24^ Thus, from the XPS analysis, we confirm and claim the presence of multi valances/charge states of nickel in the specimen. Table 1 lists the relative atomic percentages estimated for various components of nickel and oxygen that are claimed to be present.

**Table 1:** Micro-structural analyses: binding energy (BE) and relative atomic percentages of nickel and oxygen species

Peak parameter	Ni 2p_3/2_				O 1s		
	Ni	NiO	NiOOH	Ni(OH)_2_	NiO	Ni–OH	H_2_O
B.E. (eV)	852.56	853.75	855.37	856.66	529.44	531.25	532.50
Atomic percentage (%)	5.72	35.12	36.11	23.05	46.11	36.11	17.78

**Fig. 4:**
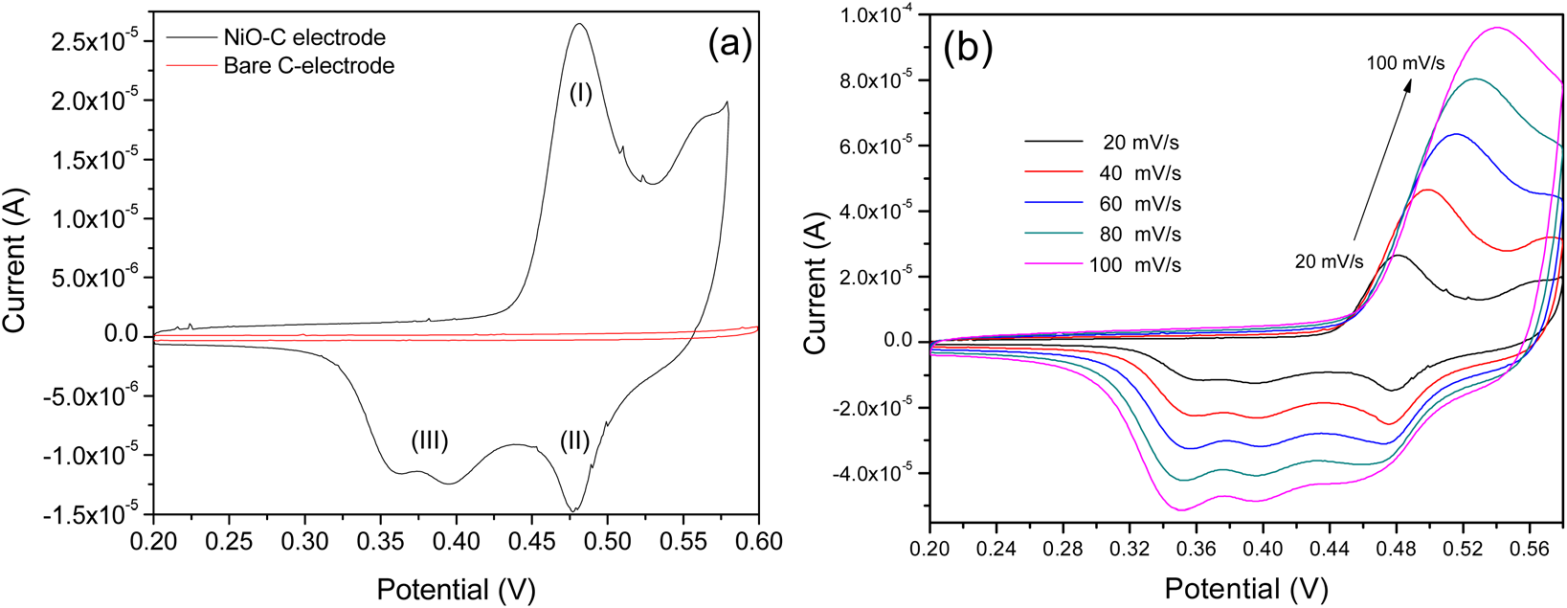
CV plot recorded for the (a) amorphous NiO_*x*_-coated screen-printed electrode and pristine screen-printed electrode at a scan rate of 20 mV s^−1^ and (b) the amorphous NiO_*x*_-coated screen-printed electrode at varying scan rates from 20 mV s^−1^ to 100 mV s^−1^.

Fig. 4 shows the comparative electrochemical responses recorded from fabricated and screen-printed electrodes in an alkaline media. The pseudocapacitive behavior associated with the fabricated electrode is clear from the CV plot (Fig. 4a) recorded at a scan rate of 20 mV s^−1^.^25^ The pseudocapacitive behavior observed herein can be attributed to the mesoporous morphology of the specimen that adsorbs OH^−^ ions present in the electrolyte. In addition, the sharp peaks observed in the CV plot indicate surface oxidation and reduction processes that occurred on the electrode. Multiple peaks observed herein are due to the multi valances of Ni that transform into oxygen and/or hydroxyl components driven by varying electrochemical potentials. It is well accepted that amorphous materials have a short range of atomic arrangement; the same is almost true for the crystallites/particles that have extremely small sizes compared to their counterparts, *i.e.* crystalline materials. The defect densities, *viz.* vacancies and trap states, associated with amorphous materials are extremely high (around 6–10 orders of magnitude higher than their crystalline counterparts). According to researchers, the reactivity and stability of such materials (in our case, NiO_*x*_) therefore result in multiple phases (in our case, enhanced Ni^2+^/Ni^3+^), which alters their transport properties; in our case, a change in cyclic voltammetry patterns was observed.^26^

In Fig. 4a, the oxidation wave (marked as ‘I’) observed in the CV plot clearly demonstrates the oxidation process occurring on the electrode surface. During the oxidation process, the transformation of Ni(ii) into multiple phases of Ni(iii), such as β-NiOOH and/or γ-NiOOH, may take place *via* the following electrochemical reactions:^27,28^

i NiO + OH^−^ ⇄ NiOOH(β) + e^−^

ii NiOOH(β) + H_2_O + e^−^ → NiOOH(γ).

According to Bode *et al.*, during the electrochemical process, NiO in an alkaline medium transforms into four phases: α-Ni(OH)_2_, β-Ni(OH)_2_, β-NiOOH and γ-NiOOH.^29^ They also confirmed that the first two phase transformations occur during the reduction process, while the latter two occur during the oxidation process.^22,23,29^ Here, it is worth noting that the formation of the NiOOH phase is essential for the effective detection of organic molecules (example glucose) present in the electrolyte. However, amongst all these phases, the presence of only β-Ni(OH)_2_ is detectable due to the highly crystalline nature associated with it according to researchers.^20,30^ Further, in the CV plot, multiple reductive waves were observed (marked as ‘II’ and ‘III’ in Fig. 4a) during the negative potential scan. The reductive peak (marked as ‘II’ in the potential range of ∼0.45–0.5 V) can be claimed as a complementary peak to the anodic one and hence is attributed to the reduction of β-NiOOH phase.^29,31,32^ A further reductive peak marked as III in Fig. 4a (observed in the potential range ∼0.325–0.375 V) indicates the reduction of γ-NiOOH to α-Ni(OH)_2_ species the following electrochemical reaction,^31^

iii NiOOH(γ) + H_2_O + e^−^ ⇌ Ni(OH)_2_(α) + OH^−^

**Fig. 5:**
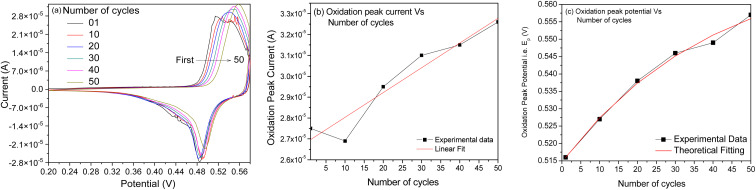
(a) CV plot recorded for the amorphous NiO_*x*_-coated screen-printed electrodes as a function of the number of cycles. (b) Plot of oxidation peak current as a function of the number of cycles. (c) Plot of oxidation peak position as a function of the number of cycles.

Since the α-Ni(OH)_2_ phase has a turbostratic structure, it accommodates extra anions, hence resulting in the broadening of particular peak width, as observed in the CV plot (Fig. 4a).^33^ Finally, the transfer of Ni(OH)_2_ species back to the NiO is anticipated *via* electrochemical-/potential-assisted decomposition process. The same CV plot (scan rate 20 mV s^−1^ and electrolyte 0.1 M NaOH) was recorded for the fabricated electrode as a function of the number of cycles, as presented in Fig. 5. With the increased number of CV scans, the oxidative peak position is observed to shift (0.51–0.56 V) during the positive potential scan, whereas the reductive peak position is observed to be fixed at one value (0.48 V) during the negative potential scan. Therefore, CV curves recorded herein were also studied in the range of 0.45–0.58 V to understand diffusion (realm as degradation, aging, phase change, stress or time-evolution of material properties) as a function of the number of cycles. To know more about the diffusion process that may take place, we calculated the apparent diffusivity, *i.e. D*_app_, as a function of the number of cycles (Fig. 5b), for which the diffusion coefficient was extracted from the CV plot recorded for each cycle (*i.e.*Fig. 5a) using the following Randles–Sevcik expression:^34^

iv *I*_p_ = 2.69 × 10^5^·*n*^3/2^·*A*·*D*^1/2^·*ν*^1/2^·*C*,

where *I*_p_, *n*, *C* and *A* represent the oxidation peak current, electron number in the rate determining step (1), the electrolyte concentration (1 × 10^−4^ mol cm^−3^), and the electrode surface area (7.1 mm^2^), respectively.

A linear fit to the database indicates that diffusivity increases linearly with the number of cycles. The diffusivity coefficient ∼7 × 10^−10^ cm^2^ s^−1^ calculated for cycles, *viz.* 1–50, indicates that surface activation or roughening associated with the electroactive area may generate unstable and/or multiple phases of Ni during the oxidation cycles.

Fig. 5c shows the variation observed in the oxidation potential peak position as a function of the number of cycles estimated from Fig. 5a (hardly any changes were observed with the reduction peak position as a function of the number of cycles; hence, this is not discussed herein). In electrochemical systems, where the surface or interfacial properties gradually stabilize due to surface conditioning/activation, adsorption/desorption balance, and passivation, the layered structures are less likely to form. Therefore, the oxidation potential *E*_p_ can be tied to the surface concentration of the active species, and the charge transfer kinetics occur on the electrode surface. The situation can be expressed using the following modified Nernst–Butler–Volmer kinetic equation:^35–39^v
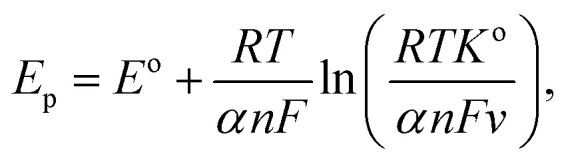
where *E*_p_ is the oxidation peak potential, which is a function of the number of cycles (*N*), *E*^o^ is the steady-state oxidation potential; *k*^o^ is the heterogeneous rate constant; *v* is the scan rate, *F* is Faraday's constant; *n* is the number of electrons transferred; and *α* is the charge transfer coefficient. Assuming that the change observed in oxidation potential is driven by adsorbate surface coverage, *i.e. θ*(*N*), which is expressed as *θ*(*N*) = 1 − e^−*kN*^, the oxidation potential *E*_p_ can be expressed using the first-order rate law as follows:vi
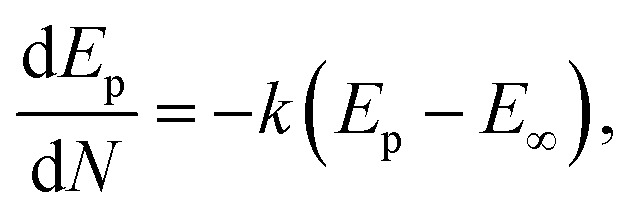
where *E*_∞_ is the asymptotic peak potential observed after many cycles and *k* is the rate constant. Solving the above differential equation by defining *N*_o_ = 1/*k*, *i.e.* the characteristic number of cycles, one can get the exponential form expressed as follows:

vii *E*_p_(*N*) = *E*_∞_ − (*E*_∞_ − *E*_1_)e^−*N*/*N*_o_^.

The form of this equation can thus often be used to study surface poisoning and/or catalyst activation. The experimental data were fitted using the above equation, as depicted in Fig. 5c. The best fit (*i.e.* goodness-of-fit (*R*^2^) = 0.994) observed herein implies that (a) the change in oxidation peak potential saturates rapidly during repeated cycling and (b) the surface modifications occurring with the electrode material can correspond to the first-order process and gradually change due to the formation of multiple phases, *i.e.* the formation of various nickel hydroxide species (as already discussed).

**Fig. 6:**
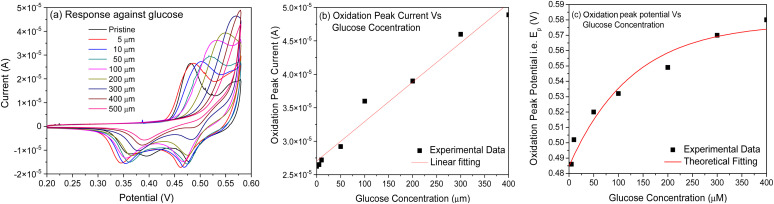
(a) CV plot recorded for the amorphous NiO_*x*_-coated screen-printed electrode as a function of glucose concentration in the electrolyte. (b) Plot of oxidation current (*I*_p_) as a function of glucose concentration. (c) Plot of oxidation peak position (*i.e. E*_p_) as a function of glucose concentration.

To confirm the electrochemical response of amorphous NiO_*x*_ coated on the screen-printed electrode against the glucose molecule, CV plots (Fig. 6a) were recorded with a fixed scan rate at 20 mV s^−1^ in the electrolyte (0.1 M NaOH) and varying glucose concentrations from 0 to 500 µM. The active nature of the electrode in terms of (a) shifts associated with the oxidation and reduction peaks and (b) changes in the area under the curves as a function of increased glucose concentration in the electrolyte is clear from the plots. The changes observed herein suggest the presence of enough amount of NiOOH to oxidize the glucose molecule *via* the following chemical reactions:^40,41^

viii NiO + OH^−^ ⇄ NiOOH + e^−^

ix NiOOH + glucose → NiO + H_2_O_2_ + gluconolactone

x gluconolactone + H_2_O → gluconate + H^+^.

With increased glucose concentration changes, the oxidation peak current and shifts in its position are observed during the positive potential scan. Therefore, once again, the CV curves (Fig. 6a) recorded herein were studied in the range of 0.45–0.58 V to understand the diffusion phenomenon as a function of glucose concentration in the electrolyte. Fig. 6b shows the plot of the oxidation peak current as a function of the glucose concentration in the electrolyte. The diffusion coefficient for the electrode was extracted using the Randles–Sevcik expression, *i.e.*eqn (iv), from the plot. The value of the diffusion coefficient estimated by fitting the database with the linear fit is observed to be 6.082 × 10^−10^ cm^2^ s^−1^, which are comparable to the typical literature values for metal oxide systems measured by varying glucose concentration in an aqueous alkaline solution.^14,30,42^ This clearly indicates the possibility of charge transfer on the electrode surface rather than contributing to an increase in current through the electrode. This must be the reason for the negligible change in current values observed with an increased glucose concentration in our case. To confirm any such charge transfer that may take place on the surface of an electrode, shifts observed with the oxidation peak position (*E*_p_) as a function of glucose concentration were extracted from the CV plots (Fig. 6a) presented in Fig. 6c. It is clear from the plot that the oxidation peak potential shifts positively with an increase in the glucose concentration in an alkaline medium. Considering the system under investigation as reversible in nature, where the oxidation peak potential (*E*_p_) dominates as a function of the electrolyte concentration (*C*), the reactions occurring on the electrode surface may follow the Nernst equation of equilibrium given below:xi
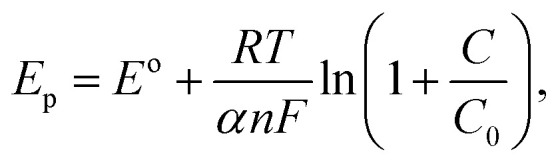
where *E*^o^ is the steady-state oxidation potential, *α* is the transfer coefficient, *F* is Faraday's constant, *n* is the number of electrons transferred, *R* = 8.314 J mol^−1^ K^−1^ and *T* is the temperature. An excellent fit to the database (Fig. 6c) using the above equation also confirms the charge transfer kinetics that occur on the electrode surface.

**Fig. 7:**
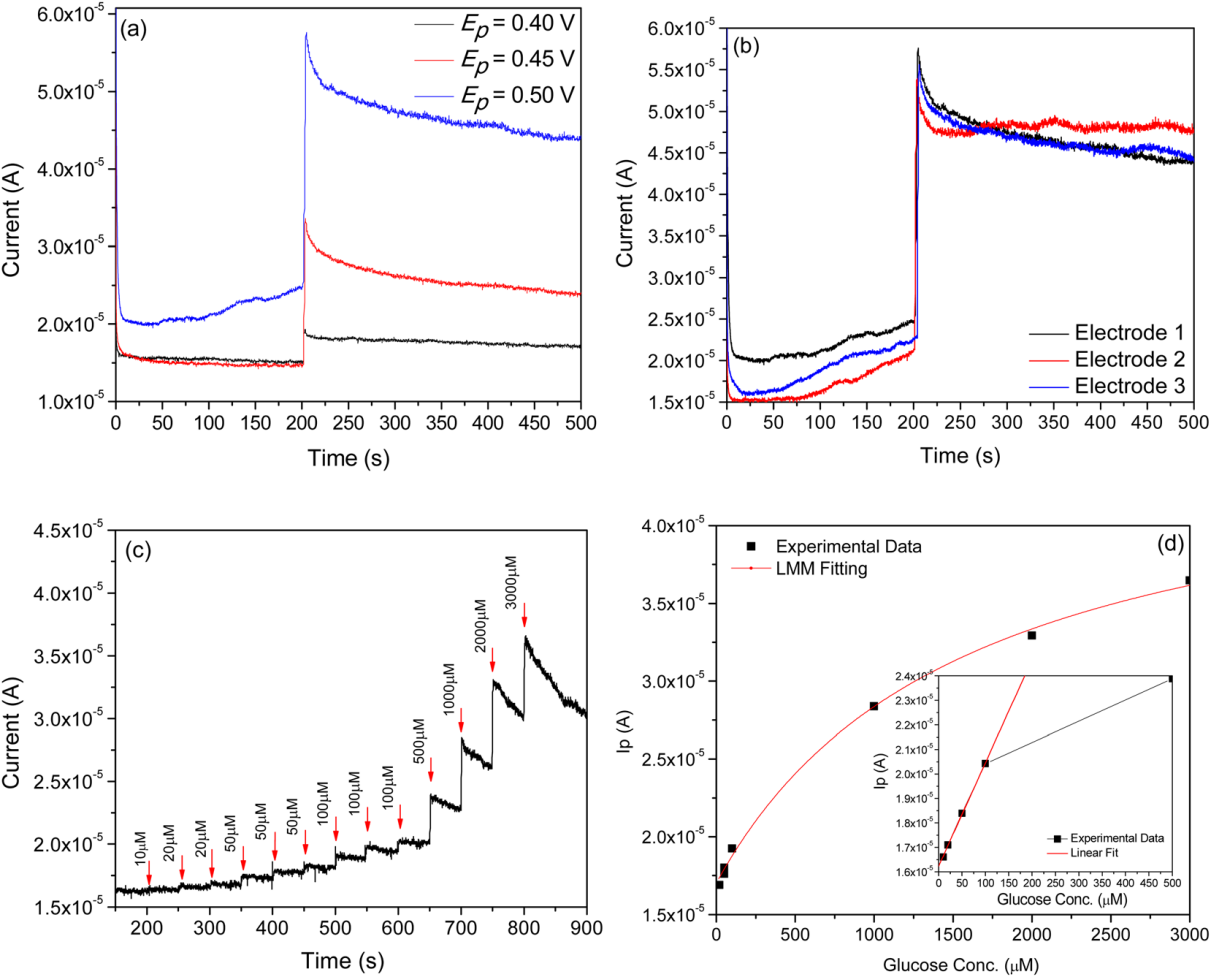
Amperometric responses of electrode to the subsequent addition of 1 mM glucose in an alkaline medium (0.1 M NaOH) (a) at different applied potentials, (b) on different electrodes applying a potential of 0.5 V to confirm consistency with the fabrication of electrodes, (c) at varying glucose concentrations (10–3000 µM) at 0.5 V in a 0.1 M NaOH solution, and (d) the respective calibration plot.

To attain an optimal amperometric response toward the analyte glucose, the response of the electrode against different applied potentials (namely 0.40, 0.45 and 0.50 V) was recorded, as presented in Fig. 7a. For this, a moderate concentration of glucose (1 mM) present in an alkaline medium (0.1 M NaOH) as an electrolyte was used. The highest sensitivity value was obtained at 0.5 V. For consistency of the the electrode/material, the experiment was repeated in the same environment on three different electrodes fabricated under the same deposition conditions. From the responses recorded on these electrodes at applied potentials of 0.5 V (*i.e.*Fig. 7b), one can confirm the consistency/repeatability with the fabrication process followed to make these electrodes.

Fig. 7c shows the amperometric responses of an electrode against varying glucose concentrations (10–3000 µM) in 0.1 M NaOH solution, recorded at a constant applied potential of +0.5 V *vs.* SCE. From the plot, it is clear that after the injection of each aliquot of glucose (each 50 s), the electrode is observed to exhibit a quick step wise increase in oxidation current response (*i.e. I*_p_) and acquires saturation within 3–4 seconds, clearly demonstrating fast kinetics of the electron transfer process. A calibration plot (Fig. 7d) derived from Fig. 7c was used to estimate the sensitivity and detection limit of the electrode. From the plot, it is clear that two distinct regions are observed. The lower glucose concentration region (10–100 µM) is where a linear relationship between oxidation current and glucose concentration is observed. The linear behavior observed herein suggests that diffusion-controlled kinetics may occur on the electrode surface. In the second region, *i.e.* the higher glucose concentration region (100–3000 µM), current saturation at high glucose concentration is observed. This behavior indicates the saturation of all catalytic/active sites on the electrode surface. Additionally, Table 2 demonstrates the NiO_*x*_ superior performance in comparison to existing sensors. To exactly simulate the situation, we decided to use the Langmuir–Michaelis–Menten (LMM) model, as it captures the adsorption-limited as well as saturation behavior of an electrode during biomolecule sensing. The model is expressed as follows:^43^xii
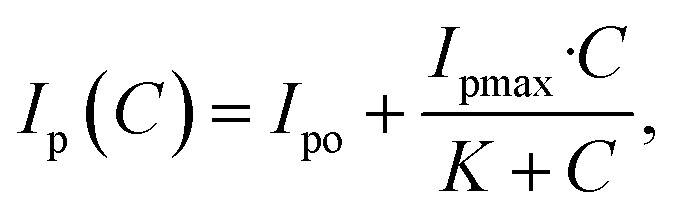
where *I*_p_(*C*) is the oxidation current at the glucose concentration, *I*_po_ is the oxidation current without the addition of glucose, *I*_pmax_ is the saturated oxidation current, and *K* is the half-saturation constant. The high goodness of fitting, *i.e. R*^2^ (∼0.995), observed herein clearly indicates that the electrode follows a clear saturation with increased glucose concentration and offers stability. Further, it is observed to be highly sensitive in the low-concentration region (∼1–10 µM). The sensitivity measured in the low concentration region (10–100 µM), *i.e.* inset in Fig. 7d, is observed to be 1.39 × 10^−8^ A µM^−1^ and has a limit of detection (LOD) ∼0.083 µM.

**Table 2:** Performance-based comparison of different sensors

Electrode system	Potential window (V)	Sensitivity	Linear range (mm)	Limit of detection (µm)	Response time (s)	Applied potential (V)	Ref.
NiO_x_	0–0.6	1.39 × 10^−8^ A µM^−1^	0.001–7	∼0.083	<3	0.5	This work
NiO	0.1–0.7	265 µA mM^−1^ cm^−2^	0.1–1	1.00	3	0.45	44
ZnO tetrapods	−1.5–1.5	29 µA mM^−1^ cm^−2^	0.05–0.7	17	—	−0.32 and −0.24	45
NiCo-LDH/MWCNTs/GCE	−0.2–0.6	2.55 µA mM^−1^ cm^−2^	0.1–9	0.03	—	0.5	46
Ni/NiO/NC/GCE	0.3–0.6	76.03 µA mM^−1^ cm^−2^	0.006–8.6	0.2	—	0.5	47
NiO/SPCE	0–0.6	1.842 µA mM^−1^	0.1–5	0.025 mM	—	0.5	48
NiO/MWCNT composite	−0.6–0.7	∼484 µA mM^−1^ cm^−2^	0.005–3	∼0.17	∼5 s	0.6	49
ALD NiO/SCCNTs	0–0.9	1252.3 µA mM^−1^ cm^−2^	0.002–2.2	0.1	<2	0.65	50
NiO nanowalls	0–0.7	2.3 mA mm^−1^ cm^−2^	0.0002–1	0.2	—	0.5	51
NiCo-LDH@Ag NWs/SPE	0–0.7	0.52 µA µM^−1^ cm^2^	0.005–4.5	1.38	7	0.45	52

## Conclusion

Amorphous NiO_*x*_ was deposited on the commercially available screen-printed electrode using a state-of-the-art technique, *i.e.* PLD. The multi valence nature of Ni associated with NiO_*x*_ grown herein was confirmed using XPS. The electrochemical activity of the electrode against glucose molecules present in an alkaline medium was investigated by recording CV plots. Negligible changes in current values and a systematic change with the oxidation potential *E*_p_ in the CV plot as a function of glucose variation were observed. The value of the diffusion coefficient was estimated from the experimental database fitted with the Randles–Sevcik expression. Negligible change in current values and systematic change with the oxidation potential *E*_p_ observed in the CV plot as a function of glucose concentration in alkaline medium indicate a charge transfer process and/or generation of the surface concentration of active species/phases on the electrode surface. We believe that the surface concentration of active species/phases, namely α-Ni(OH)_2_, β-Ni(OH)_2_, β-NiOOH and γ-NiOOH, generated on the electrode surface drives various electrochemical reactions, which are responsible for glucose oxidation, followed by its detection. Investigations based on the theoretical models suggest that the surface modifications occurring on the electrode surface are first-order processes, which gradually change due to the formation of various nickel hydroxide species.

## Conflicts of interest

There is no actual or potential conflict of interest in relation to this article.

## Data Availability

The data will be made available on request.
